# Progression of diabetic retinopathy during pregnancy in a woman with ABCC8-MODY

**DOI:** 10.1530/EDM-25-0149

**Published:** 2026-02-11

**Authors:** Patrick McCluskey, Mairéad T Crowley, Cosmina Barac, Maria Kennelly, Maria M Byrne

**Affiliations:** ^1^ Department of Endocrinology, Mater Misericordiae University Hospital, Dublin, Ireland; ^2^ Department of Ophthalmology, Mater Misericordiae University Hospital, Dublin, Ireland; ^3^ Department of Obstetrics and Gynaecology, Rotunda Maternity Hospital, Dublin, Ireland

**Keywords:** diabetes, rare diseases/syndromes, reproduction

## Abstract

**Summary:**

Activating mutations in the *ABCC8* gene are extremely rare and cause ABCC8-MODY. The phenotype is variable with onset of diabetes in childhood/early adulthood. Retinopathy is the most common reported complication. We describe a 31-year-old primigravida woman referred to and seen at our antenatal ambulatory diabetes clinic at 6 weeks plus 5 days gestation. She had a strong family history of diabetes and was diagnosed at the age of 11 years. Genetic testing revealed an activating pathogenic c.4139G>A variant in the *ABCC8* gene. She was managed with glibenclamide, sitagliptin, and dapagliflozin. Her complications included mild bilateral non-proliferative retinopathy and necrobiosis lipoidica. Her BMI was 19 kg/m^2^, and HbA1c was 68 mmol/mol. Oral agents were discontinued, and insulin therapy was commenced. At 22 weeks gestation, routine retinal screening identified progression to bilateral active proliferative diabetic retinopathy (time in range for pregnancy (TIRp) 68%, HbA1c 39 mmol/mol). She received four sessions of panretinal photocoagulation (PRP) bilaterally between 22 and 33 weeks gestation. There was no associated loss of vision or nephropathy. TIRp was ≥70% for the remainder of the pregnancy. She delivered a 3.9 kg unaffected female infant at 38 weeks via elective caesarean section without maternal or neonatal complications. Bilateral active proliferative retinopathy persisted up to 61 weeks postnatally and required additional PRP. Forty-six weeks post-partum, after ceasing breastfeeding, insulin was switched to glibenclamide and dapagliflozin. This is the first case report of rapid progression of clinically significant diabetic retinopathy during pregnancy in a woman with ABCC8-MODY. This is an unusual finding as there is a relatively low risk of significant progression of diabetic retinopathy in women with type 2 diabetes during pregnancy.

**Learning points:**

- Rapid progression of diabetic retinopathy during pregnancy is an uncommon complication in women with type 2 diabetes.
- This woman with ABCC8-MODY developed rapidly progressive clinically significant diabetic retinopathy without nephropathy during pregnancy.
- There is a paucity of literature regarding pregnancy-related complications of ABCC8-MODY.
- This case highlights the risks of end organ disease in a rare form of diabetes.

## Background

Activating mutations in *ABCC8* result in the development of ABCC8 maturity-onset diabetes of the young (ABCC8-MODY), which is a very rare form of autosomal dominant monogenic diabetes, transient neonatal diabetes, or permanent neonatal diabetes (1). *ABCC8* encodes an isomer of the ATP-binding cassette (ABC) protein, sulphonylurea receptor 1 (SUR1), which is expressed in the pancreatic beta cells, in brain cells, in all the layers of human retina, and in the endothelial cells of retinal vessels (2, 3). Activating mutations prevent closure of the potassium channels in pancreatic beta cells, resulting in reduced insulin secretion and the development of diabetes. ABCC8-MODY responds well to low-dose sulphonylurea (SU) therapy (3, 4).

The prevalence of micro- and macro-vascular complications in ABCC8-MODY remains unknown; however, previous studies have shown that retinopathy is the most common complication and may occur with rapid progression (3, 5, 6). It is well described that there is a relatively low risk of significant progression of diabetic retinopathy in pregnant women with type 2 diabetes (7). Herein, we present the case of rapidly progressive diabetic retinopathy in a pregnant woman with ABCC8-MODY.

## Case presentation

**Figure 1:**
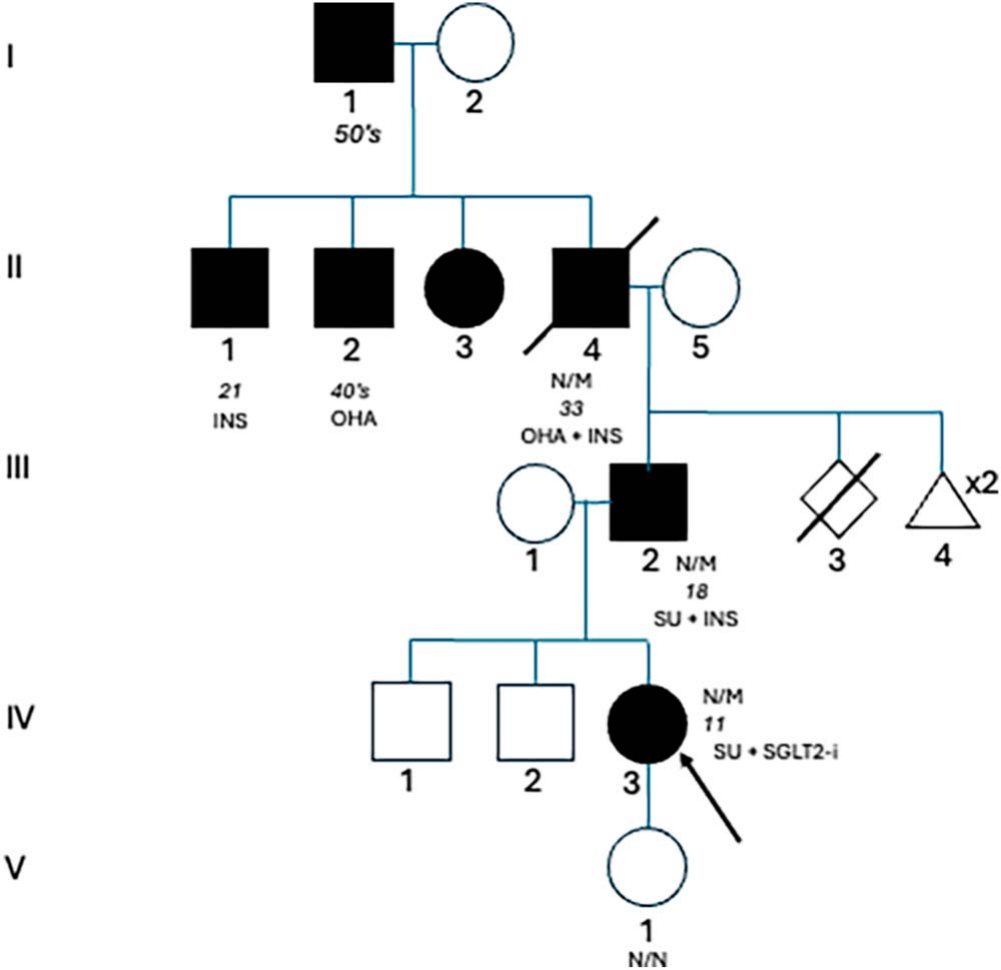
Pedigree of patient’s family adapted from Bowman *et al.* (1). The squares represent male sex, and the circles represent female sex. The black shading indicates diabetes phenotype. Genotype, where known, is displayed under symbols. N/M indicates heterozygosity for *ABCC8* c.4139G>A, p.(Arg1380His). N/N indicates no pathogenic variant identified. Age (italics) at diagnosis and treatment are indicated below genotypes. OHA, oral hypoglycaemic agent; SU, sulphonylurea; SGLT2i, sodium/glucose cotransporter 2 inhibitor; INS, insulin. The patient is the proband and is indicated by an arrow.

A 31-year-old primigravida woman was referred to and seen at our antenatal ambulatory diabetes clinic at 6 weeks plus 5 days gestation. Her personal birth weight was 3.3 kg at 42 weeks gestation, with no history of neonatal dysglycaemia. She was diagnosed with diabetes at 11 years of age and had a strong family history of diabetes and was diagnosed with an activating pathogenic c.4139G>A, p.(Arg1380His) variant in the *ABCC8* gene shown in a previous case series (1). The family fulfilled MODY criteria in terms of age at onset of diabetes less than 25 years in at least one family member and the presence of autosomal dominant pattern of inheritance. Her father was diagnosed at age 18, and he was on SU and insulin at age 61 years. Her paternal grandfather was diagnosed at age 33 and was on SU and on insulin for the last 5 years of life. The adapted pedigree is shown in Fig. 1 (1). She was managed with diet and lifestyle modifications and subsequently initiated on glibenclamide at age 14 years. At 18 years, her BMI was 19 kg/m^2^ and HbA1c was 75–86 mmol/mol on repaglinide 2 mg po tds. At the age of 25 years, she was changed to glibenclamide 10 mg bd and sitagliptin 100 mg od. One year later, her HbA1c was 84 mmol/mol, and she failed the addition of metformin 500 mg od due to gastrointestinal side effects. Dapagliflozin 10 mg od was added at age 28 years. She achieved a HbA1c of 53 mmol/mol on these three agents. Her diabetic complications included bilateral mild non-proliferative diabetic retinopathy and necrobiosis lipoidica on her left shin. She had no evidence of diabetic neuropathy, or nephropathy and no history of hypertension. There was no family history of retinopathy, neonatal dysglycaemia, schooling difficulties, or neurodevelopmental disorders.

**Figure 2:**
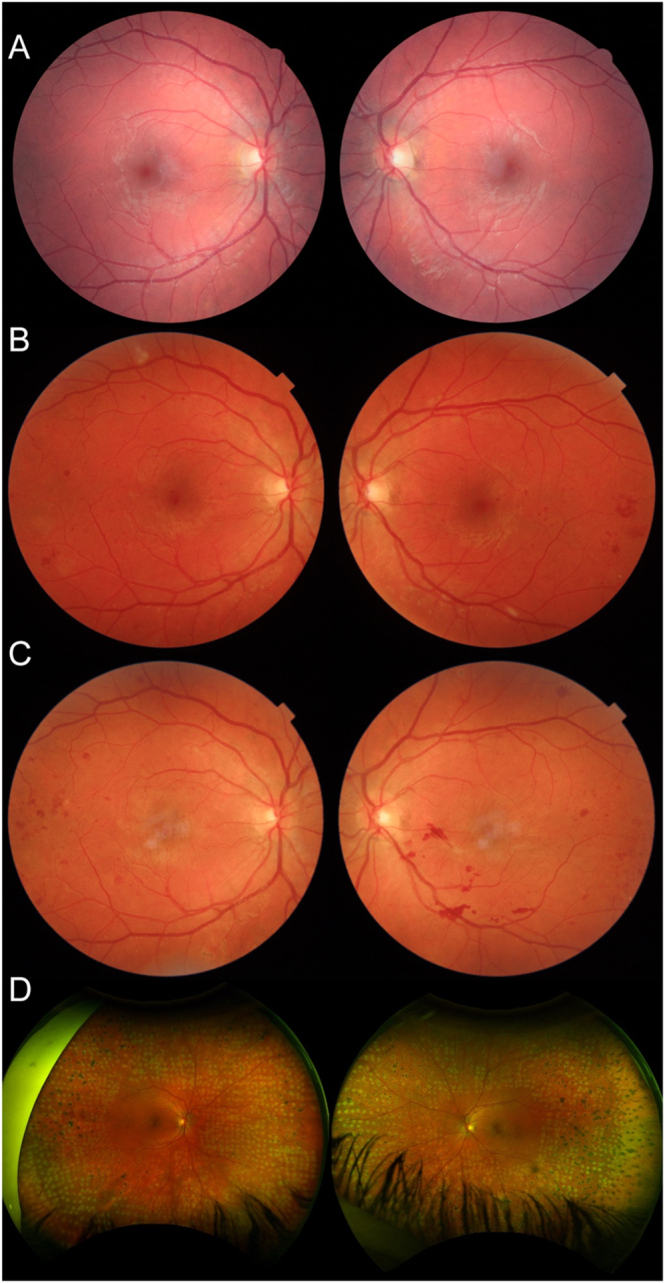
(A) Retinal imaging 8 years pre-pregnancy demonstrating bilateral mild non-proliferative diabetic retinopathy. (B) Retinal imaging at 22 weeks gestation showing bilateral active proliferative retinopathy. (C) Retinal imaging at 3 weeks post-partum showing active proliferative retinopathy despite bilateral PRP. (D) Imaging of right and left eye at 61 weeks post-partum demonstrating bilateral active proliferative diabetic retinopathy and fresh laser marks from the same-day PRP treatment.

At presentation to the combined antenatal diabetes clinic, she was normotensive and had a BMI of 24 kg/m^2^. Her most recent retinal screening showed bilateral mild non-proliferative retinopathy (Fig. 2A). She had no evidence of nephropathy or neuropathy and had no history of cardiovascular disease. Her oral antihyperglycaemics were changed to insulin aspart and insulin isophane with an initial total daily dose (TDD) of 29 units/day. Her HbA1c was 68 mmol/mol, corrected fructosamine 397 μmol/L, creatinine 47 μmol/L, and urine protein:creatinine ratio 9 g/mol (normal limit < 30). Her fasting capillary blood glucose readings were between 8.7 and 10.1 mmol/L. She had no other past medical history and was on no other medications.

At 22 weeks gestation, routine retinal screening raised high suspicion for bilateral active proliferative diabetic retinopathy (Fig. 2B). Following this, urgent eye examination and treatment were arranged in the local diabetic retina treatment clinic. The patient received four sessions of panretinal photocoagulation (PRP) in each eye. There was no proteinuria on urinalysis, and she was normotensive with a blood pressure of 111/74 mmHg. Fetal growth scan at 31 weeks showed large for gestational age with an abdominal circumference >95th centile. At 33 weeks gestation, she had a mild vitreous haemorrhage with subsequent resolution after fill-in PRP. While she had diabetic maculopathy, she did not have any macular oedema and maintained excellent vision at all time points.

**Figure 3:**
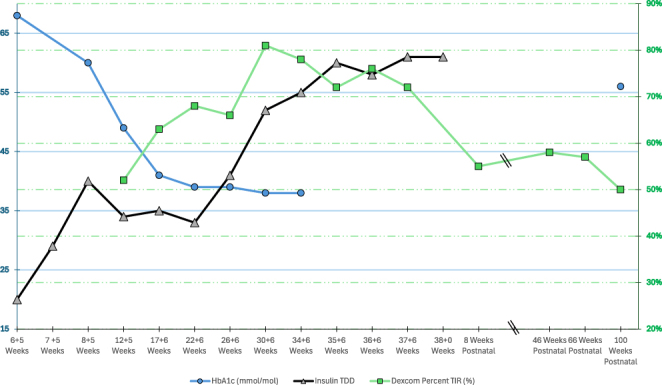
HbA1c (mmol/mol), TIRp (% between 3.5 and 7.8 mmol/L) on Dexcom G7 CGM, and TDD insulin (units/day) plotted versus weeks of gestation and post-partum. Postnatal TIR (% between 3.9 and 10 mmol/L).

Her glycaemic control remained on target during her third trimester with a TIRp between 72 and 81%, HbA1c 38 mmol/mol (5.6%), and corrected fructosamine between 271 and 321 μmol/L (Fig. 3). At 38 weeks gestation, she had an elective caesarean section delivering a 3.9 kg female infant (UK-WHO growth chart birth centile: 91st) with no neonatal hypoglycaemia. Genetic testing on the infant was negative for the c.4139G>A variant.

## Investigation

A Dexcom G7 continuous glucose monitor (CGM) was started at 9 weeks gestation, and TIRp and HbA1c throughout the pregnancy are shown in Fig. 3. At 12 + 5 weeks gestation, her HbA1c was 49 mmol/mol and her TIRp (3.5–7.8 mmol/L) was 52% (4% very high, 42% high, 1% low, and <1% very low). At 22 weeks, her TIRp was 68% (0% very high, 31% high, 1% low, and 0% very low). At 38 weeks and at the time of delivery, her TIRp was 73% (0% very high, 26% high, 1% low, and <1% very low). In pregnancy, the target is a TIRp > 70%.

Serial fetal growth scans with reference to a standard Irish population showed an abdominal circumference in the 78th centile at 21 weeks, 59th centile at 24 weeks, >95th centile at 31 weeks, and >95th centile at 35 weeks. Retinal screening via Topcon and Optos wide-field imaging at 22 weeks gestation identified bilateral active proliferative diabetic retinopathy and maculopathy without macular oedema. Repeat retinal imaging at 33 weeks gestation showed a mild vitreous haemorrhage and continued to demonstrate bilateral active proliferative retinopathy.

## Treatment

At her initial visit, she was started on insulin aspart and insulin isophane subcutaneously with an initial TDD of 29 units/day. Her median (quartile) TDD was 35 (34–37) units in the second trimester and 59 (56–61) units in the third trimester. Changes to insulin therapy were made with the aim of maintaining glycaemic control TIRp above 70% with target range defined as glucose 3.5–7.8 mmol/L. TDD insulin (units/day) versus weeks of gestation and post-partum is shown in Fig. 3.

She initially had four sessions of panretinal photocoagulation (PRP) in each eye between 22 and 33 weeks gestation. At 33 weeks gestation, she had fill-in PRP after identification of a mild vitreous haemorrhage, which subsequently resolved.

## Outcome and follow-up

Post-partum, she was continued on insulin with a TDD of 34 units. After the patient discontinued breastfeeding at 46 weeks postnatally, her insulin was stopped and she was restarted on glibenclamide 12.5 mg/5 mg morning and evening, respectively, and dapagliflozin 10 mg od. More recently, 23 months post-partum, she was maintained on glibenclamide 10 mg/7.5 mg morning/evening and dapagliflozin 10 mg od with a TIR 50% and HbA1c 56 mmol/mol.

Retinal imaging at 3 weeks postnatally continued to demonstrate active proliferative retinopathy despite bilateral PRP during pregnancy (Fig. 2C). Further retinal imaging at 66 weeks postnatally continued to demonstrate bilateral active proliferative retinopathy, which required further PRP (Fig. 2D). Her vision remained excellent at last review, and she continues not to display evidence of nephropathy or neuropathy.

The infant who tested negative for the c.4139G>A variant remains well and has reached all developmental milestones.

## Discussion

This case is the first description of the unusual onset of rapidly progressive diabetic retinopathy in a pregnant woman with ABCC8-MODY. She had pre-existing risk factors for the development of diabetic retinopathy, including a 20-year duration of diabetes and a history of suboptimal glycaemic control. In addition, her presenting HbA1c was significantly elevated. However, it is well described that there is a relatively low risk of significant progression of diabetic retinopathy in women with type 2 diabetes, which is in sharp contrast to type 1 diabetes where up to 17% of women progress to significant retinopathy during pregnancy (7). Given that our patient did not have type 1 diabetes, it was unusual for her to develop clinically significant retinopathy in the pregnancy. In addition, rapid correction of HbA1c in individuals with type 2 diabetes results in significantly less progression of diabetic retinopathy compared to that seen in type 1 diabetes (8, 9).

Diabetes in pregnancy is associated with a number of serious maternal and fetal complications. The presence of pre-existing diabetic retinopathy and rapid correction of hyperglycaemia are possible contributing factors for the progression of retinopathy in our case. Women planning pregnancy should have pre-conception review of diabetes and complications with informed discussion on the risk of worsening or occurrence of complications during pregnancy (10). Current guidelines recommend that, prior to conception, medications should be changed to those considered safer in pregnancy and glycaemic control should be optimized to achieve the guideline target of HbA1c < 48 mmol/mol. In addition, women with the c.4139G>A variant should be counselled that the fetus has a 50% chance of inheriting this gain-of-function mutation (also described as ‘activating’), which may result in diabetes that can be diagnosed in the neonatal period (at age <6 months) or in childhood/early adulthood as MODY (3). During pregnancy, HbA1c should be targeted to <42 mmol/mol with fasting glucose 3.9–5.3 mmol/L and 1-h postprandial glucose 6.1–7.8 mmol/L (10). Dilated eye examination should occur ideally before pregnancy as well as in the first trimester and then every trimester and for 1 year post-partum.

ABCC8-MODY may confer an increased risk of developing diabetic retinopathy compared to other forms of MODY diabetes. In our previous small cohort study of 10 individuals from four families with gain-of-function *ABCC8* variants, half had evidence of diabetic retinopathy, of which only one had an additional microvascular manifestation of diabetes and one individual (c.2497G>A) on insulin displayed rapid progression of diabetic retinopathy (3). This development of rapid progression of diabetic retinopathy has also been reported in another case study of a 27-year-old male on insulin with ABCC8-MODY [p.(Ala1457Thr)] (6). There has additionally been a large genomic study of an American Indian population where the loss-of-function *ABCC8* p.(Arg1420His) variant was associated with a significantly higher odds ratio for the development of diabetic retinopathy with and without adjustment for fasting blood glucose; notably, treatment modality at any point with SU, insulin, metformin, and thiazolidinedione lacked significant association with the presence of diabetic retinopathy (11). There are also other reports of early advanced complications of ABCC8-MODY, including a Charcot’s foot in a 35-year-old female with a pathogenic c.3544C>T p.(Arg1182Trp) variant and severe nephrotic syndrome with end-stage renal disease requiring haemodialysis in a 25-year-old male with the c.1616A>G p.(Tyr539Cys) variant treated with insulin (5, 12). Large phenotypical variability occurs within pedigrees with ABCC8 mutations.

Older porcine and rat *in vitro* studies have demonstrated the SU, glibenclamide, inhibits adenosine-mediated retinal vasodilation at subtherapeutic concentrations. This was reinforced with an *in vivo* study in both acute excitotoxicity and type 2 diabetes Goto–Kakizaki rat models with the administration of subtherapeutic doses of intravitreal glibenclamide, resulting in an improvement of the features associated with diabetic retinopathy. The protective effect of glibenclamide was lost when SUR1 expression was suppressed by 44.6% with siRNA pre-treatment directed against SUR1; altogether, this suggests a retinal-protective effect of glibenclamide mediated through SUR1 binding that is independent of glycaemic changes. The study further demonstrated that glibenclamide, likely through binding to SUR1, resulted in the upregulation of 300 genes, including significant upregulation in genes known to harbour neuroprotective and antioxidant effects (2).

While pregnancy and rapid correction of hyperglycaemia may have contributed to the rapid development of clinically significant retinopathy, we would suggest that there is a theoretical possibility that the resultant change in SUR1 due to the *ABCC8* pathological variant may contribute to the rapid progression to proliferative retinopathy. Her glycaemic control was suboptimal on glibenclamide 10 mg bd; thus, insulin therapy was required to achieve optimal glycaemic control during pregnancy. Despite optimization of glycaemic control, her infant was large for gestational age, probably secondary to suboptimal control during the first trimester. Our subject was treated with insulin during the pregnancy although one could consider continued SU use in *ABCC8* pregnancies; there are no reports of SU usage administration during *ABCC8* pregnancies to date. However, a recent publication described improved neurodevelopment following *in utero* SU exposure in a patient with *KCNJ11* permanent neonatal diabetes which affects another subunit of the pacreatic beta cell ATP-sensitive potssium channel (13). The variant in our case is not known to be associated with permanent neonatal diabetes.

Unfortunately, due to the lack of widespread reporting of the complications of affected individuals in the literature, it remains difficult to make definitive conclusions about the prevalence of diabetic complications in ABCC8-MODY and there is a need for further investment in the recruitment and study of affected individuals. In addition, while some individuals with pathogenic *ABCC8* variants have poor response to SU, many are very responsive, and the possibility raised by animal studies of retinal-protective effects of SU warrants closer examination in individuals with ABCC8-MODY. This case highlights the importance of genetic testing to establish accurate diagnosis and allow for precision medicine.

## Declaration of interest

The authors declare that there is no conflict of interest that could be perceived as prejudicing the impartiality of the work reported.

## Funding

This research did not receive any specific grant form any funding agency in the public, commercial, or not-for-profit sector.

## Patient consent

Written informed consent for the publication of the patient’s clinical details and clinical images was obtained from the patient.

## Author contribution statement

PMcC and MB researched the case, reviewed the existing literature, and drafted the manuscript. MTC, CB, and MK were involved in the patient’s care and reviewed and edited the manuscript. MB is the guarantor of this work and, as such, had full access to all the data in the study and takes responsibility for the integrity of the data.
